# Plume divergence characterization for electric propulsion via standard deviation and emittance

**DOI:** 10.1007/s44205-025-00113-5

**Published:** 2025-02-25

**Authors:** McKenna J. D. Breddan, Richard E. Wirz

**Affiliations:** Mechanical and Aerospace Engineering, University of California, los Angeles, 420 Portola Plaza, Los Angeles, 90095 CA USA

**Keywords:** Electric propulsion, Divergence, Emittance, Electrospray

## Abstract

Electric propulsion systems require careful consideration of plume divergence and evolution over a range of operating conditions and environments. Existing means of describing plume divergence such as outlines, plume profiles, and snapshots of the plume are dominated by outlier particles and do not provide reliable or quantitative insight. Proposed herein are two novel methods for describing plume divergence using *standard deviation* and *emittance* to provide quantitative insight of the collective behavior of plume species. Furthermore, the emittance metric from the particle accelerator community is shown to accurately describe plume evolution in a two-dimensional position and momentum angle space. Cross-sectional emittance measurements are used to display the presence of non-Hamiltonian forces in plume evolution, namely stochastic Coulomb collisions between neighboring particles. Finally, full-plume emittance diagrams are demonstrated as a means of identifying when an electric propulsion plume has reached steady state.

## Introduction

Electric propulsion (EP) systems offer very high specific impulse, creating more thrust per unit of propellant mass than chemical propulsion systems. This capability allows them to enable deep space [1, 2] and small satellite missions [3, 4], as well as perform attitude control and station keeping [5–8]. The primary life-limiting mechanism in many electric propulsion systems is the erosion of electrodes [9–11] or channel walls [12, 13]. The divergence of the plume within the thruster system directly impacts the erosion of internal thruster surfaces, such as electrodes and channel walls, with propellant particles [9, 10, 14]. Additionally, the plume outside of the thruster system impacts the interaction of the plume with external components of the spacecraft [8, 15]. Recently, EP researchers have been tackling the significant challenge of the facility effects of background pressure in ground test facilities on measured thruster plume behavior and divergence [16–20]. While divergence is a key challenge for the lifetime, performance, and facility effects of electric propulsion systems, the community has yet to converge on a consistent quantitative method for characterizing the divergence of the plumes produced by these systems.

Traditionally, to find the thrust correction, $T_{corr}$, due to plume divergence for a given charged species it is recommended to integrate over the plume profile near the thruster exit by

1 $$\begin{aligned} T_{corr,i} = \frac{\int _0^r{2 \pi r j_i(r) \cos [\theta (r)] dr}}{J_{B,i}}, \end{aligned}$$

where, for each plume species, *i*, the value $j_i (r)$ is the local current density at position *r* with angle $\theta (r)$ normalized by the total beam current $J_{B,i}$. In this way, for each species, *i*, an effective divergence, $\theta _{eff,i}$, can be found by $T_{corr,i} = \cos (\theta _{eff,i})$. This approach can be expanded to include all species such that

2 $$\begin{aligned} \theta _{eff} = \textrm{arccos}{\left( \frac{\mathcal {T}_{act}}{\mathcal {T}_{ideal}}\right) }, \end{aligned}$$

where, $\mathcal {T}_{ideal}$, is the ideal thrust without divergence and $\mathcal {T}_{act}$ is the actual thrust which can be measured or summed over all species with thrust correction by

3 $$\begin{aligned} \mathcal {T}_{act} = \sum \limits _i^n{\mathcal {T}_{ideal,i} T_{corr,i}}. \end{aligned}$$

This approach represents a commonly used method for using beam divergence for thrust correction but does not capture the higher angles represented by species of the plume that, as discussed earlier, can cause deleterious interactions with spacecraft, thruster, and facility surfaces. Additionally, this method does not provide insight into plume evolution or impacts of environmental and facility effects. Therefore, it is important to also capture the divergence more holistically by the methods proposed herein.

Current methods for presenting simulated or experimentally observed electric propulsion plume divergence in the literature include plume outlines [21, 22], two-dimensional snapshots of the plume [21–24], and plume profiles [25, 26]. Plume outlines are dictated by outlier particles, which can reach much wider angles than the majority of plume particles. Two-dimensional snapshots of the plume visually represent all particles rather than solely the widest particles, but are not quantifiable. Experimental plume profiles are time-averaged over an observation window and are thus limited in the temporal resolution with which they can describe plume evolution and divergence. Furthermore, plume profiles do not provide a quantitative value for divergence without specifying a reference point on the profile, such as the widest observed angle. The objective of this publication is to propose a definition for the divergence of electric propulsion plumes which is quantifiable and representative of the collective behavior of the plume species, rather than outlier particles. Furthermore, recognizing that plumes can evolve in momentum in addition to position, emittance [27–32] is proposed as a quantitative electric propulsion plume divergence metric which accounts for momentum evolution in addition to positional evolution.

## Methods

We propose two methods for divergence characterization: $3\sigma$ standard deviation and emittance. This publication characterizes these methods by investigating the divergence of an electrospray plume as an example of a plume produced by an electric propulsion system. The methods presented herein are widely applicable to plumes produced by other types of electric propulsion systems.

### $3\sigma$ plume divergence

**Fig. 1:**
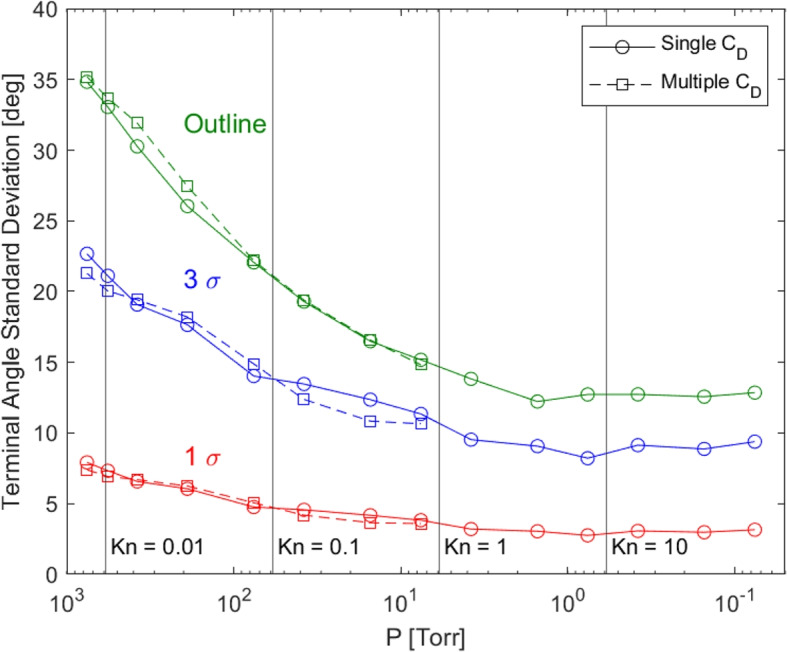
Terminal angle measurements at the collector plate location are given for 1 and 3 standard deviations of particle number density and the outline of plumes simulated at different background pressures using a single coefficient of drag from Loth et al. [33] (solid lines) and multiple coefficients of drag from Stokes [34], Moshfegh et al. [35], Niazmand et al. [36], and Tao et al. [37] depending on particle *Re* and *Kn* (dashed lines), from Breddan and Wirz [17]

Existing means of presenting plume divergence include plume outlines, plume profiles, and 2-dimensional snapshots of the plume. The plume outline is dictated by outlier particles and can therefore overstate the divergence of the majority of the plume. In contrast, we propose using the $3\sigma$ boundary to provide a more statistically relevant representation of beam divergence. Figure 1 presents the terminal angles on the downstream collector plate reached by one standard deviation ($1\sigma$, containing 68.27%), three standard deviations ($3 \sigma$, containing 99.73%), and the outline (containing 100%) of particle number density for various background pressures. The standard deviation measurements were obtained by dividing the plumes axially into sections and radially fitting a Gaussian profile to particle number density distributions in each section. Other distributions, such as a a Super-Gaussian fit, could also be used in cases for which they fit better to the particle mass density distribution [25].

For EP plumes, the terminal angle, $\theta$, is defined as the polar angle measurement,

4 $$\begin{aligned} \theta = \textrm{tan}\left( \frac{\sqrt{x^2 + y^2}}{z}\right) , \end{aligned}$$

where *x*, *y*, and *z* are the 3D positional coordinates upon reaching the collector plate at the downstream end of the simulation domain, or the effective terminal surface in an EP facility. As shown in Fig. 1, there is a significant angular difference between the $3\sigma$ and outline measurements, especially at high background pressure levels where collisions cause plume expansion [33]. By definition, the $3\sigma$ representation captures the angle within which we find 99.73% of the plume species. For the plume shown in Fig. 1, we see that the outlier particles, those beyond $3\sigma$, account for a large range of wide incidence angles but only represent 0.27% of particles. Therefore, utilizing the outline to represent plume divergence overstates the divergence of the bulk of the plume, while using an approach such as the $3\sigma$ representation provides a better overall description of the plume’s divergence and potential for meaningful interaction with surfaces at higher angles. As described in Thuppul et al. [9], these highest angle species can be important for life but must be considered quantitatively instead of the binary cataloging of impinging or non-impinging plumes.

**Fig. 2:**
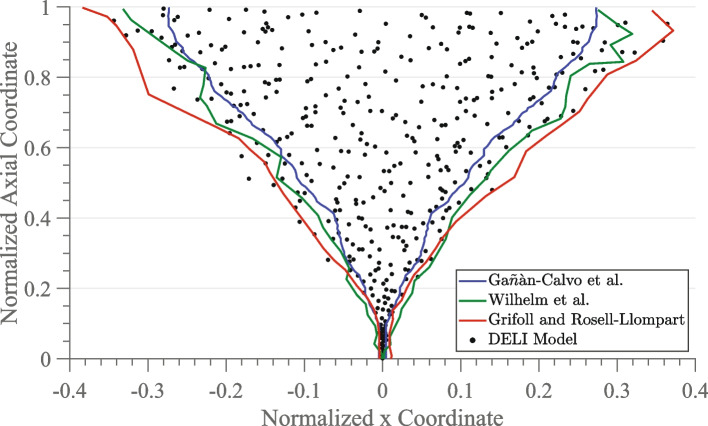
A 2-D instantaneous snapshot of the steady-state plume simulated with the UCLA Discrete Electrospray Lagrangian Interaction Model is compared with previously published outline results for the same plume, from Breddan and Wirz [21]

Snapshots of the plume, such as that presented in Fig. 2, are not determined solely by outlier particles, but rather represent every particle in the plume. However, such snapshots are not quantifiable. One can utilize plume snapshots, or compare snapshots to outlines, to visually compare plume divergence as presented in Fig. 2, but it is not feasible to quantify plume divergence with such snapshots. Therefore, while snapshots are a means of visualizing plume divergence, they are not a plume divergence metric. Similarly, plume profiles [25, 26] present a means of visualizing plume divergence, but do not quantify divergence and as such are not a plume divergence metric. Plume profiles can be used to quantity plume divergence only if a reference point on the profile is identified at which to measure angular divergence.

In some beam applications, the outermost angle of the profile is the best representation of divergence angle. In a focused beam like a laser, the angular divergence from the emission axis is defined based on the widest particles in the beam:

5 $$\begin{aligned} \theta = \textrm{arctan}\left( \frac{\Delta r_b}{l}\right) \end{aligned}$$

where $\Delta r_b$ is the change beam radius (measured from the beam center to the outline containing 100% of particles) and *l* is the distance traveled along the primary axis of beam motion. Fundamentally, electrospray plumes and the plumes or beams produced by other electric propulsion systems [38–41] are collections of moving particles, similar to a laser beam; however, plumes produced by electric propulsion systems are diffuse and have no sharp edge. As presented in Fig. 1, the plume outline set by the widest particle trajectories is much broader than most particle trajectories, such that outlines misrepresent the divergence of the bulk of the plume. Therefore, this publication proposes that the effective plume ‘edge’ for electric propulsion systems can be defined based on three standard deviations, $3\sigma$, of a Gaussian fit to the particle mass density distribution. Three standard deviations represents the statistical bulk (99.73%) of the plume without being skewed by statistically insignificant outlier particle trajectories. Particle mass density is the key metric for characterizing electric propulsion plume divergence because thruster lifetime is limited by the mass of propellant incident on downstream grids or channel walls [9–13]. Plume divergence angle at distance *z* downstream of emission is thereby obtained from Eq. 5 by substituting the radius of three of standard deviations of particle mass density, $\Delta r_{3\sigma }$, for beam edge, $\Delta r_b$, and *z* for the path length, *l*:

6 $$\begin{aligned} \theta _{3\sigma } = \textrm{arctan}\left( \frac{\Delta r_{3\sigma }}{z}\right) . \end{aligned}$$

Utilizing Fig. 1 as an example, following Eq. 6 the electrospray plume has divergence of 23.5$^{\circ }$ at atmospheric pressure ($P =$ 760Torr). Based on the application or objective of a particular effort, one may choose to use higher or lower standard deviations. For example, if grid impingement of less than 0.27% of the plume leads to significant erosion or the loss of the accelerator system via contamination (e.g. Thuppul et al. [9]) one may choose a higher standard deviation.

### Plume divergence analysis via emittance

Beam emittance, $\epsilon$, provides a powerful and quantitative method for characterizing electric propulsion plume divergence and evolution. If used properly, emittance, which is a beam parallelism measure from the particle accelerator community [27–32, 42, 43], can be used in the near-thruster region to characterize the impacts of inter- and intra-species collisions in the plume as well as expansion and propagation behavior in the vacuum facility and spacecraft environments. Accelerator beams such as the Large Hadron Collider (LHC) at CERN propagate within confining systems for kilometers [44], orders of magnitude longer distances than the beams and plumes produced by electric propulsion systems which propagate relatively short distances within the thruster, mm to cm, and order of meters within a test facility or spacecraft environment. In pursuit of keeping charged particle beams steady and confined for such long distances, the particle accelerator research community has developed a rich literature of beam quality analysis and diagnostic metrics [27–32, 42, 43]. Emittance is one such beam quality metric, which displays the influence of non-Hamiltonian forces on the beam. Emittance plots convey a transverse particle position component against the same component of transverse angle, where angle in this context [27–32, 42, 43] is defined as the ratio of momentum in the transverse direction to momentum in the axial direction of beam propagation,

7 $$\begin{aligned} x' = \frac{p_x}{p_z}. \end{aligned}$$

A quantitative emittance metric for a collection of particles can be calculated using the area of the particle collection in the position-angle trace space for a transverse component direction; the emittance in the *x*-direction is

8 $$\begin{aligned} \epsilon _x = \frac{1}{\pi } \int \int dx dx'. \end{aligned}$$

Liouville’s Theorem of emittance conservation states that emittance is conserved in a non-accelerating beam with no Hamiltonian forces [28, 42, 43]. If the beam is being accelerated, the conserved property is normalized emittance, $\epsilon _n$, such that

9 $$\begin{aligned} \epsilon _n = \epsilon \beta \gamma \end{aligned}$$

where Lorentz parameters $\beta$ and $\gamma$ are defined by

10 $$\begin{aligned} \beta = \frac{v}{c}, \end{aligned}$$

where *v* is particle velocity and *c* is the speed of light, and

11 $$\begin{aligned} \gamma = \frac{1}{\sqrt{1-\beta ^2}}. \end{aligned}$$

**Fig. 3:**
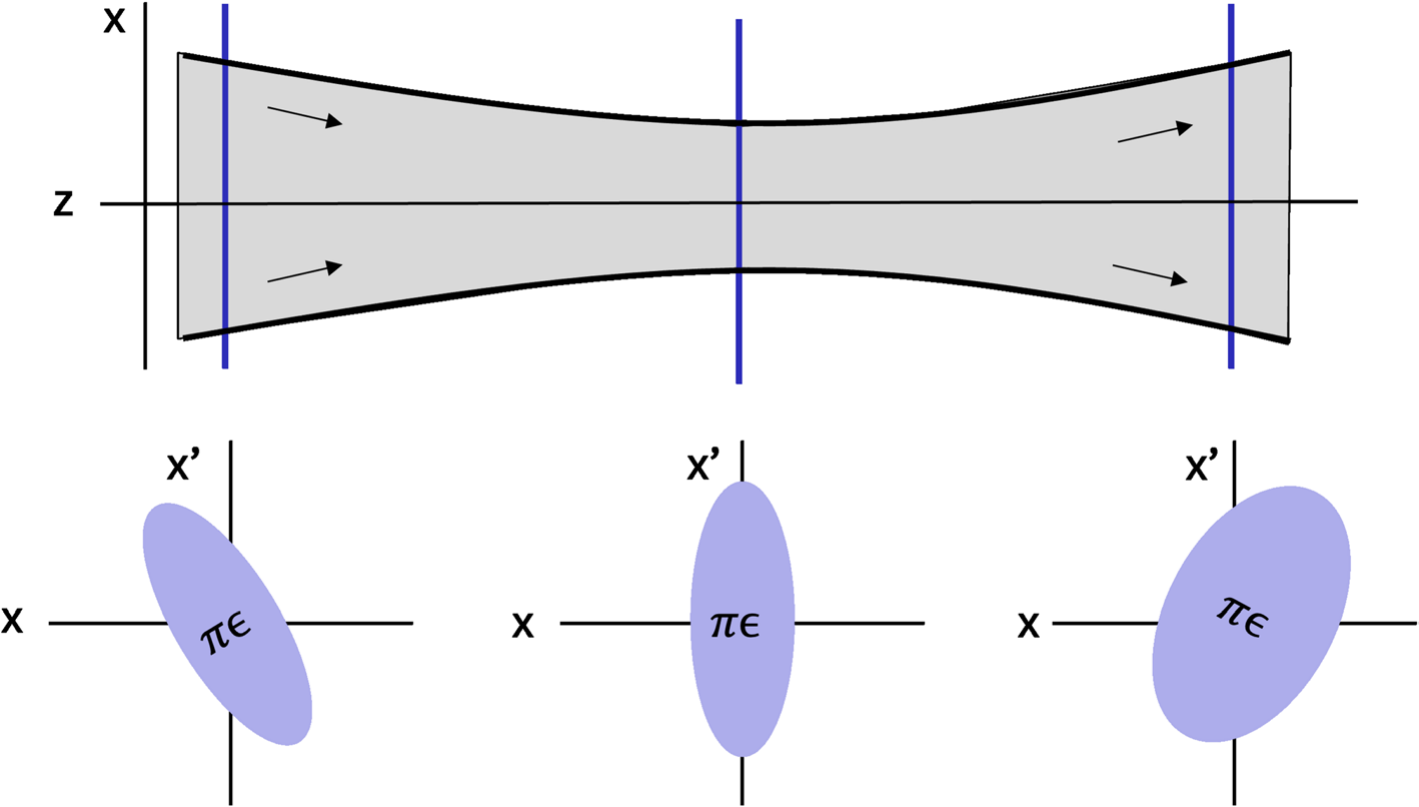
Cross sections of the plume (blue lines) are used to observe trends in emittance as the plume moves downstream

Emittance decreases in response to non-Hamiltonian forces like cooling, and increases in response to non-Hamiltonian forces such as heating, friction, scattering phenomena [45], or close-range Coulomb ‘collisions’ termed ‘intra-beam scattering’ in the CERN literature [27, 28]. The overall space charge of a charged plume creates an electric potential field which exerts Hamiltonian forces on particles and does not increase emittance; however, the stochastic, close-range Coulomb ‘collisions’ between neighboring particles do not create a consistent Hamiltonian force potential field and therefore do increase emittance. Such Coulomb collisions violate Liouville’s Theorem of emittance conservation in 6-dimension phase space (3 dimensions of position and 3 of momentum) by initiating Markov processes separate from the Hamiltonian influence of the space charge of the overall plume [28]. Close-range Coulomb interactions between charged particles are an inherent, dominant force in the plume divergence of electrosprays and other electric propulsion systems [21, 46, 47], such that intra-beam scattering is an inherent source of emittance growth in electric propulsion plumes. Emittance evolution in a beam is observed by taking emittance measurements of cross-sectional beam slices as demonstrated in Fig. 3.

In Fig. 3, a beam converges and then diverges. The emittance plots of the cross-sectional particle distributions display an elliptical shape conventional for propagating beams [29]. When the beam is converging or diverging, the particles with the widest *x* position have the highest magnitude of momentum angle $x'$. The sign of the momentum angle $x'$ is negative when particles are moving downwards in the negative *x* direction and positive in the opposite direction, such that converging beams correspond to a backwards-leaning ellipse, and diverging beams correspond to a forward-leaning ellipse. The beam waist is identifiable by the emittance plot with the lowest range in the horizontal coordinate *x*. The emittance is calculated following Eq. 8 by dividing volume of the minimum bounding ellipse of particles in the position-momentum trace space by $\pi$. Emittance in Fig. 3 can be seen to remain constant between the first two cross-sections, conveying that the beam is not accelerating and or influenced by non-Hamiltonian forces in this region. However, between the second and third cross-sections, emittance grows, signifying the presence of non-Hamiltonian forces such as the close range Coulomb forces involved in intra-beam scattering.

## Results and discussion

Plume simulation results presented in this publication were obtained by the Plasma, Energy & Space Propulsion Laboratory (PESPL) at the University of California, Los Angeles (UCLA) using the Discrete Electrospray Lagrangian Interaction (DELI) Model. The algorithm details and model validation have been published previously [21]. The electrospray geometry, 1.13nl/s flowrate constraint, and emitted EMI-Im particle data for the presented plume divergence results are from Miller et al. [48]. Three species of EMI-Im particles were utilized with the properties presented in Table 1.

**Table 1:** Tri-species EMI-Im electrospray mass and integer charge number properties [48]. All particles are negatively charged

Attribute	Small species	Medium species	Large species
Mass [amu]	9.99e6	5.99.e7	2.78e8
Charge Number	90	252	650

**Fig. 4:**
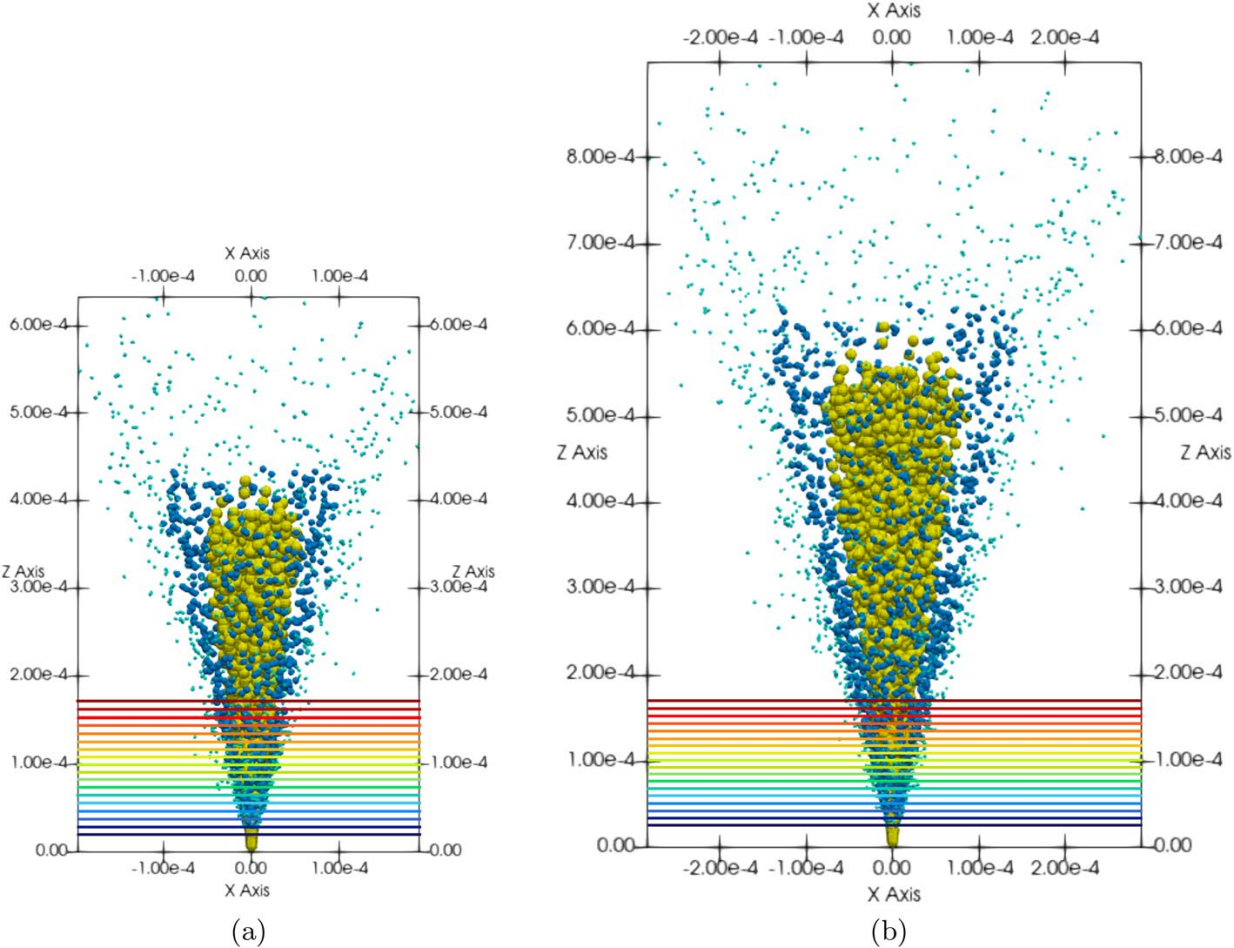
Cross sections of the tri-species EMI-Im plume at which emittance measurements were taken **a** 100 $\upmu$s and **b** 150 $\upmu$s into simulation time

Fifty simulation frames of particle data taken 1 $\upmu$s timesteps apart were averaged for the presented emittance analysis, beginning 100 $\upmu$s into the simulation time. Figure 4 presents a 2-D $x-z$ snapshot of the electrospray plume at the beginning and end of this emittance analysis window. The cross-sectional beam slices at which emittance measurements were taken are shown as lines across the plume with color corresponding to axial coordinate. Particles were considered in the emittance analysis of a cross section if they were within 0.5 $\upmu$m of the cross-section line. Computational limitations prevent simulating this EMI-Im plume to steady state within the full 1.5 mm simulation domain; therefore, the plume is in a startup period and reaches further downstream in each timestep. To restrict the emittance analysis to a steady portion of the plume with high particle density in all analyzed timesteps, only the lower portion of the plume was considered in the presented emittance study, as shown in Fig. 4. While there are particles present above this analysis region even at the start of the analysis period, that upper portion of the plume expands further in future timesteps. Therefore, only the lower portion of the plume which is steady across all timesteps is considered in this time-averaged emittance analysis.

**Fig. 5:**
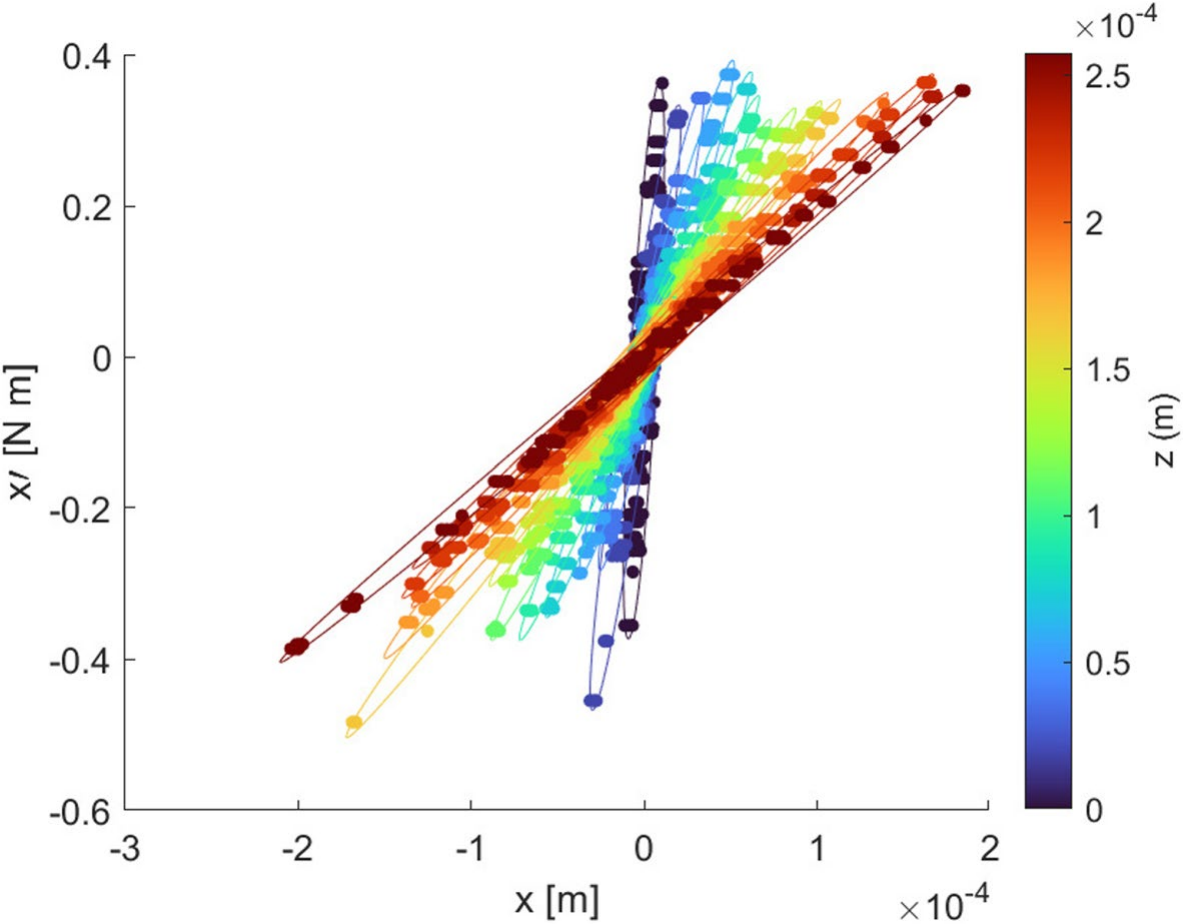
Emittance plots of the particles from each cross-sectional beam slice in Fig. 4 100 $\upmu$s into simulation time

The time-averaged emittance plots at each cross-section of the plume are presented in Fig. 5, with color corresponding to axial coordinate of the cross-section as in Fig. 4. As displayed in the diverging beam example in Fig. 3, the emittance ellipses become forward-leaning as the plume propagates downstream, signaling divergence. This forward lean in the ellipses is created by a simultaneous increase in lateral positional coordinate and decrease in lateral momentum angle. The lateral momentum angle $x'$ of each particle can be reduced by particle mass to yield velocity angle,

12 $$\begin{aligned} v_{x}' = \frac{v_x}{v_z}. \end{aligned}$$

Therefore, the emittance plot displays a decrease in velocity angle as the particles move downstream. This result fits with the understanding of Coulomb-driven plume divergence [21, 23, 49], in which close-range Coulomb interactions cause significant divergence in the charge-dense emission region, whereas particles further downstream in less charge dense regions experience less divergence as they are guided by electric field lines.

**Fig. 6:**
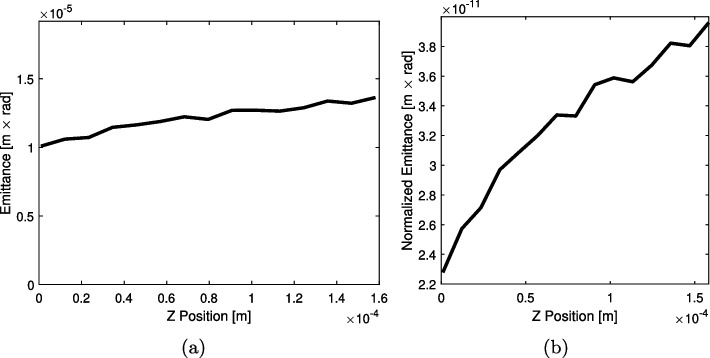
**a** Emittance and **b** normalized emittance evolution in the *x* component direction against axial coordinate. Each data point is an emittance measurement at a cross-section of the plume

The emittance and normalized emittance at each cross section presented in Fig. 5 were calculated using the area of the minimum enclosing ellipses following Eqs. 8 and 9, respectively. This process was repeated for each timestep in the emittance analysis period, and the emittance and normalized emittance results from each timestep were averaged. Figure 6 plots these time-averaged emittance results against axial coordinate to display emittance and normalized emittance evolution as the electrospray plume propagates downstream. The presented results are for the *x* component of emittance; the plume is symmetric such that the *y* component results show the same trends. Both emittance and normalized emittance can be seen to increase as the plume propagates, supporting the understanding that plume divergence is driven by stochastic Coulomb collisions between neighboring charged particles, which are a source of emittance growth. The emittance slope is less than the normalized emittance slope due to the electrostatic beam acceleration; the normalized emittance removes this acceleration effect to show solely the effects of non-Hamiltonian forces such as stochastic Coulomb collisions. While computational limits prevented the simulation of this plume further downstream in the simulation domain, the frequency of near-neighbor Coulomb collisions is known to decrease as particles move downstream of the charge-dense emission region. Therefore, the emittance and normalized emittance are expected to decrease in slope as the plume moves further downstream, eventually reaching a plateau when plume particles are too spread apart to significantly interact Coulombically and instead follow electric field lines, which is a Hamiltonian process. Future emittance evolution research will target the detection of such an emittance plateau region further downstream of emission.

**Fig. 7:**
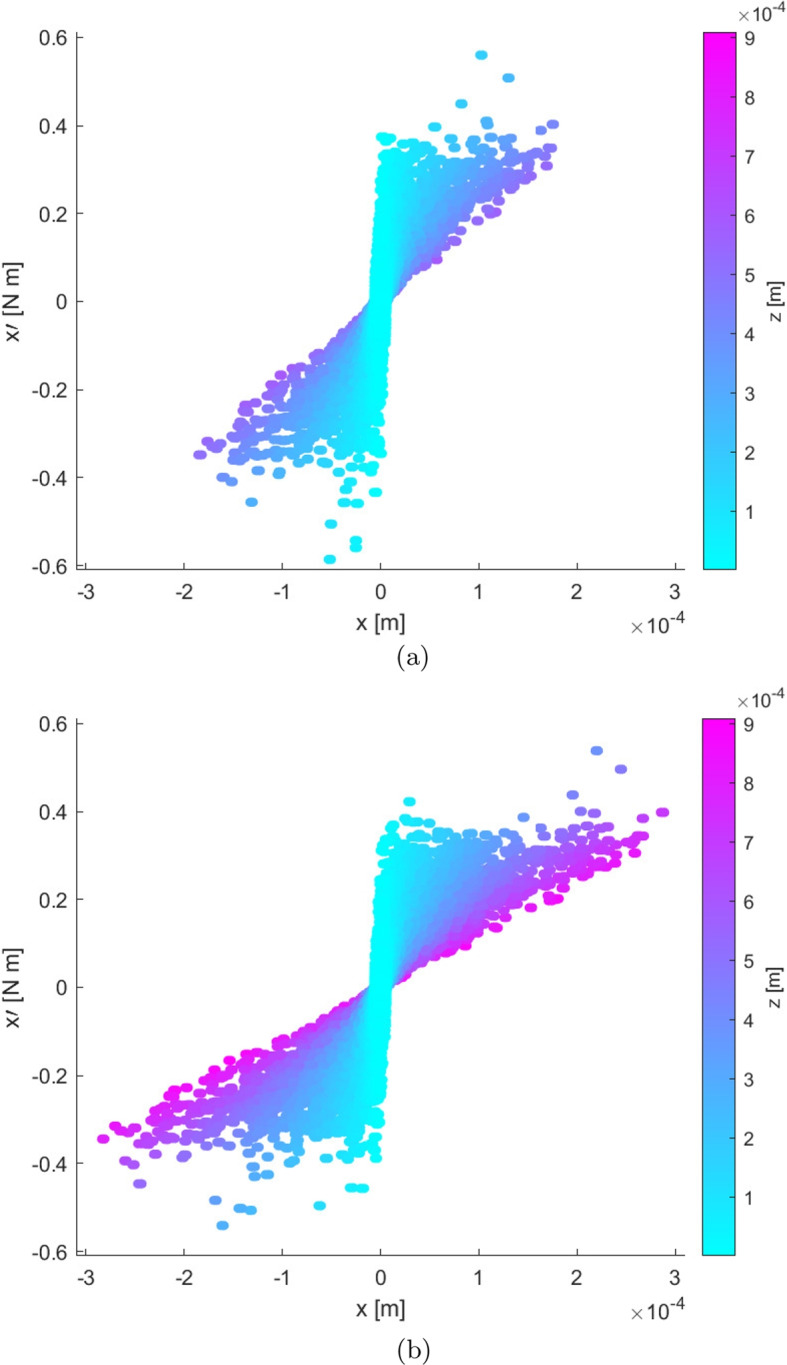
Emittance plots of all particles in the simulated tri-species EMI-Im electrospray plume at **a** 100 $\upmu$s and **b** 150 $\upmu$s of simulation time

Emittance is further useful in identifying steady state in the plumes produced by electric propulsion systems such as electrosprays. The existing method of identifying steady state in simulated electrospray plumes is observing a plateau in the number of particles in the domain [21, 24, 50]. While this metric detects steadiness in the plume population, it neglects to consider steadiness in the plume’s velocity distribution. Emittance plots, on the other hand, display both position and momentum such that an emittance plot of all particles in the plume, as opposed to a cross-sectional slice, can be used to detect steadiness in the full plume’s position and momentum angle distributions. Emittance plots of the *x*-component of all particles in the tri-species EMI-Im plume at 100 $\upmu$s and 150 $\upmu$s of simulation time are presented in Fig. 7. Color corresponds to axial coordinate as in the previous cross-sectional emittance plot; however, the color scale has been changed to reflect the full range of axial coordinates in the plume, rather than only the lower steady plume portion. The plume can clearly be seen to evolve between timesteps in the emittance plots: particles reach further downstream axially, the range of *x*-positions occupied by particles expands, and the range of *x*-momentum angles occupied by particles expands at many axial positions. In the later timestep, the emittance diagram is more forward-leaning, displaying that the plume is further diverging over time. Overall, the shape of the emittance distribution clearly varies between the time steps, showing that this plume has not reached steady state during that interval. When the full plume has reached steady state, the emittance plot will cease to show further changes over time in structure: the *x*-position, *x*-momentum angle, and axial coordinate ranges of particles will be constant, as they will be for the *y* lateral component of the plume. Therefore, this publication proposes that the plumes produced by electric propulsion systems can be defined to have reached steady state when their full-plume emittance plots become steady in shape over time. Demonstrating steady state plume detection through full-plume emittance steadiness will be completed in future research.

## Conclusion

Plume divergence is a key metric for the lifetime and performance of electric propulsion systems; however, the literature has yet to converge on common metrics for plume divergence. Existing means of conveying plume divergence, including outlines, two-dimensional plume snapshots, and plume profiles are either dominated by outlier particles or are not quantifiable metrics. In this publication, a definition for the angular divergence of electric propulsion plumes has been proposed based on three standard deviations of a Gaussian fit to the particle mass density distribution over radial coordinate:

13 $$\begin{aligned} \theta _{3\sigma } = \textrm{arctan}\left( \frac{\Delta r_{3\sigma }}{z}\right) . \end{aligned}$$

Further proposed was the utilization of the emittance metric from the particle accelerator community as a two-dimensional beam divergence metric which considers both position and momentum. Using an electrospray as an example of an electric propulsion system, cross-sectional emittance measurements in the direction of plume propagation displayed that emittance and normalized emittance grow as the plume propagates, affirming the dominance of stochastic, near-neighbor Coulomb interactions in plume evolution. The slope of emittance was found to be less than the slope of normalized emittance, as normalized emittance removes the influence of beam acceleration, which decreases emittance. Germane to EP analyses, normalized emittance removes the effect of plume acceleration and allows improved characterization of non-Hamiltonian forces such as intra- and inter-species collisions. It is hypothesized that emittance will plateau when the plume has propagated sufficiently far downstream to reach an electrostatically-dominated region beyond the ‘interaction region’ where Coulomb interactions are dominant. Bounding this interaction region is an active area of electrospray research [49–51] to which emittance can contribute a novel lens. While observing this plateau in emittance is beyond the scope of this publication due to computational limitations, this emittance plateau phenomena will be investigated in future work. Finally, emittance plots of the full plume in position-momentum angle trace space were proposed as a means of identifying when a plume has reached steady state. The full plume emittance plot continues to evolve in shape when the plume is evolving its position or momentum angle distribution, such that when the emittance plot is steady in shape, the plume has reached steady state in both its position and momentum angle distributions.

Regarding emittance for EP plume analysis, there are many opportunities to borrow additional emittance related analysis techniques from the particle accelerator community [27–32, 42, 43]. Notably, much insight can be gained from all four Courant-Snyder or Twiss parameters, which include emittance, $\epsilon$, and the ellipse orientation parameters: $\gamma$, $\alpha$, and $\beta$. For example, one can use parameters such as beam *half width*, $\sqrt{\beta \epsilon }$, and beam *half divergence*, $\sqrt{\gamma \epsilon }$ [29, 52–54]. Also, $\alpha$ can be used to describes correlations between *x* and $x'$ to indicate beam convergence or divergence. Further investigation of the correlation of emittance evolution with intra-species and inter-species (e.g. background species collisions due to facility effects) is also encouraged. The authors see these areas as an opportunities for future work for ourselves and the broader EP community.

In this study, angular divergence and emittance metrics have been applied to an electrospray plume as an example of wider applicability to the plumes produced by electric propulsion systems. These metrics are widely applicable to particle beams of any type. Therefore, fellow researchers of electric propulsion systems [47, 55–64] as well as targeted aerosols and other particle beams [65–69] are encouraged to apply these metrics to further illuminate the divergence of beams in many applications.

## Data Availability

No datasets were generated or analysed during the current study.
